# Berberine ameliorates fatty acid-induced oxidative stress in human hepatoma cells

**DOI:** 10.1038/s41598-017-11860-3

**Published:** 2017-09-12

**Authors:** Yixuan Sun, Xinlu Yuan, Feifei Zhang, Yamei Han, Xinxia Chang, Xi Xu, Yu Li, Xin Gao

**Affiliations:** 1 Department of Endocrinology and Metabolism, Zhongshan Hospital, Fudan University, Shanghai, 200032 China; 2 Fudan Institute for Metabolic Diseases, Shanghai, 200032 China; 3 CAS Key Laboratory of Nutrition and Metabolism, Institute for Nutritional Sciences, Shanghai Institutes for Biological Sciences, University of Chinese Academy of Sciences, Shanghai, 200031 China

## Abstract

Oxidative stress is thought to be critical for the pathogenesis of hepatic steatosis and its progress to non-alcoholic steatohepatitis. Berberine (BBR) can improve hepatic steatosis. In this study, we investigated the role of BBR in ameliorating oxidative stress. Lipid accumulation was measured in the livers of C57BL/6 mice fed a high fat diet (HFD) or a normal diet for 8 weeks, then either received BBR or vehicle for the study duration. Nrf2 distribution was detected in male Sprague-Dawley rats’ livers *in vivo* and in Huh7 cells *in vitro*. ROS generation and mitochondrial complex expression was measured in Huh7 cells. HepG2 cells were employed for the measurement of oxygen consumption rates. Our results showed that BBR reduced triglyceride accumulation in the liver of HFD-fed mice. The activation and nuclear distribution of Nrf2 was decreased in the hepatocytes of rats that received BBR treatment, while on a HFD. BBR also markedly reduced Nox2-dependent cytoplasmic ROS production and mitochondrial ROS production, which was mediated by the down-regulation of Complex I and III expression. In conclusion, BBR has a great potential to reduce the effects of oxidative stress, which likely contributes to its protective effect in inhibiting the progression of hepatic steatosis to steatohepatitis.

## Introduction

Non-alcoholic fatty liver disease (NAFLD) is prevalent in both developed and developing countries. NAFLD is a major health problem that can progress to non-alcoholic steatohepatitis (NASH), fibrosis, and even hepatic cancer^1^. NAFLD is also closely associated with the development of insulin resistance and type 2 diabetes, as well as cardiovascular disease^1^. Lifestyle intervention, including diet and physical exercise, can effectively improve fatty liver. However, lifestyle intervention is often insufficient, as it requires significant changes that must be maintained over the patient’s lifetime; long term compliance is not always achievable, and lifestyle changes do not necessarily reverse the damage that has occurred. Therefore, multiple agents, such as insulin sensitizers (e.g., pioglitazone) and anti-oxidants (e.g., vitamin E) have been used in patients and have been associated with improvement in the histologic findings with regard to hepatic steatosis^2, 3^. Thus, this group of drugs represents a promising avenue for the development of new NAFLD treatments. Berberine (BBR) is an isoquinoline-type alkaloid extracted from a Chinese herb, Huanglian^4^. Our previous study showed that BBR, when administered along with lifestyle intervention, reduced hepatic fat content in patients by 53% during a 16-week intervention^5^. Lipid accumulation is the first hit in a “two-hit” theory, which best describes the progression of NAFLD. The second hit involves cellular lipid overload, which provides excess substrates for oxidation, contributing to reactive oxygen species (ROS) production and oxidative stress. However, the underlying mechanism(s) by which BBR improves NAFLD endpoints in patients is not well understood. Therefore, we investigated the ability of BBR to reduce oxidative stress *in vitro* in human cells and *in vivo* in rodent models of NAFLD.

## Results

### BBR prevents high fat diet–induced NAFLD

**Figure 1:**
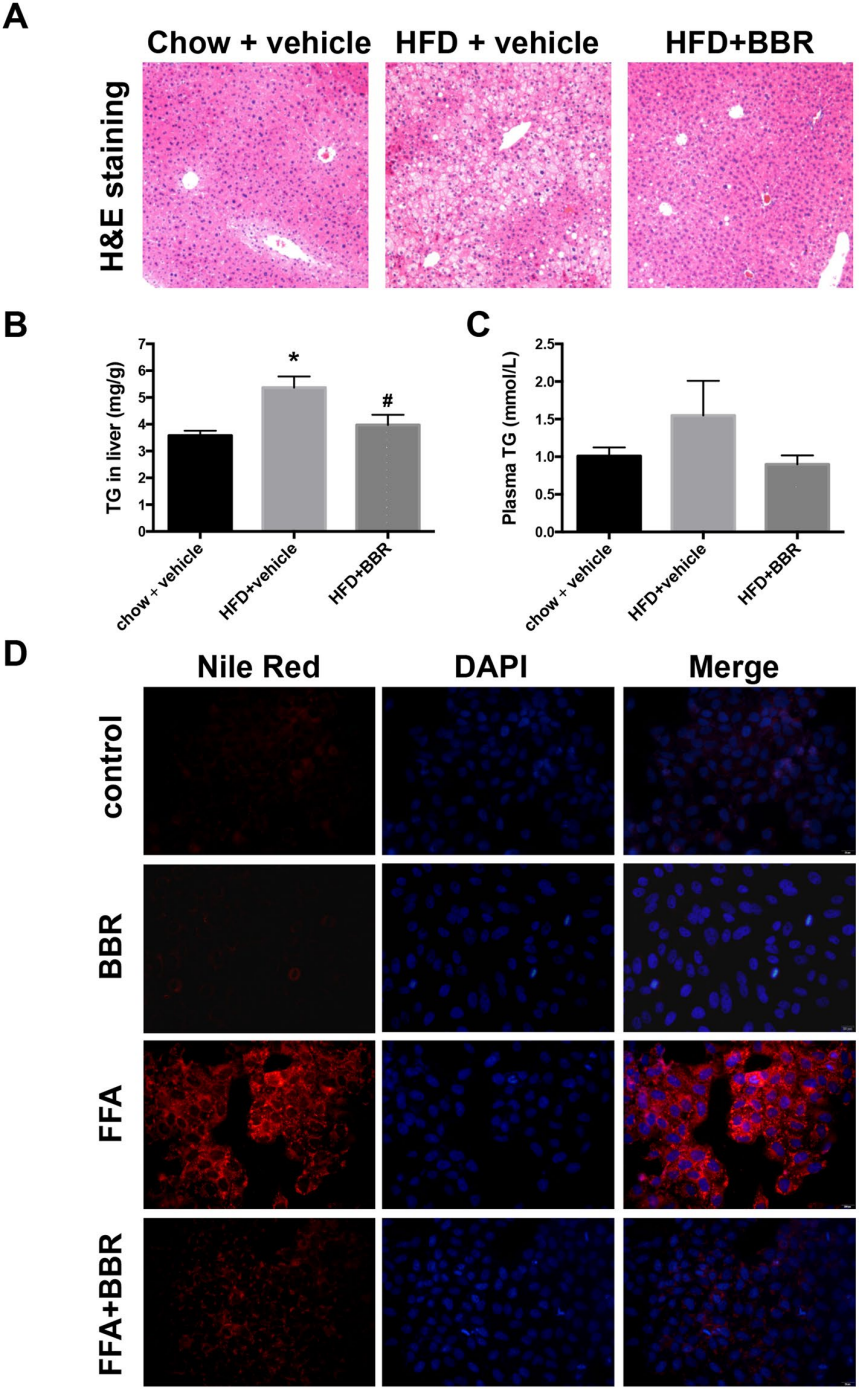
BBR reduces triglyceride accumulation in the livers of C57BL/6 mice and Huh7 cells. Male C57BL/6 mice were fed a high-fat diet (HFD) or standard chow for 16 consecutive weeks and received either BBR or vehicle for 8 weeks by gavage. The liver sections were stained by (**A**) haematoxylin and eosin (H&E), and (**B**) the hepatic triglycerides (TG) and (**C**) the TGs in the plasma were measured. (**D**) Huh7 cells were treated with free fatty acids (FFAs), BBR, and FFA+BBR. Triglycerides were determined fluorometrically using Nile red.

First, we investigated the role of BBR in the improvement of NAFLD in male C57BL/6 mice, which developed NAFLD after an 8-week high fat diet, as shown by the development of hepatic steatosis (Fig. 1A). A substantial reduction in hepatic lipid deposition was observed in HFD-fed mice that were orally administered BBR versus those administered vehicle control (Fig. 1A). These results were further supported by a change in liver triglyceride (TG) content (Fig. 1B), and there was also a slight decrease in the plasma TG levels of the HFD + BBR group compared to the HFD group (Fig. 1C). Consistent with these *in vivo* results, administration of 10 µM BBR also significantly reduced the lipid accumulation in FFA-stimulated Huh7 cells, which further confirmed the lipid-lowering effect of BBR (Fig. 1D).

### BBR inhibits the activation of the transcription factor Nrf2

**Figure 2:**
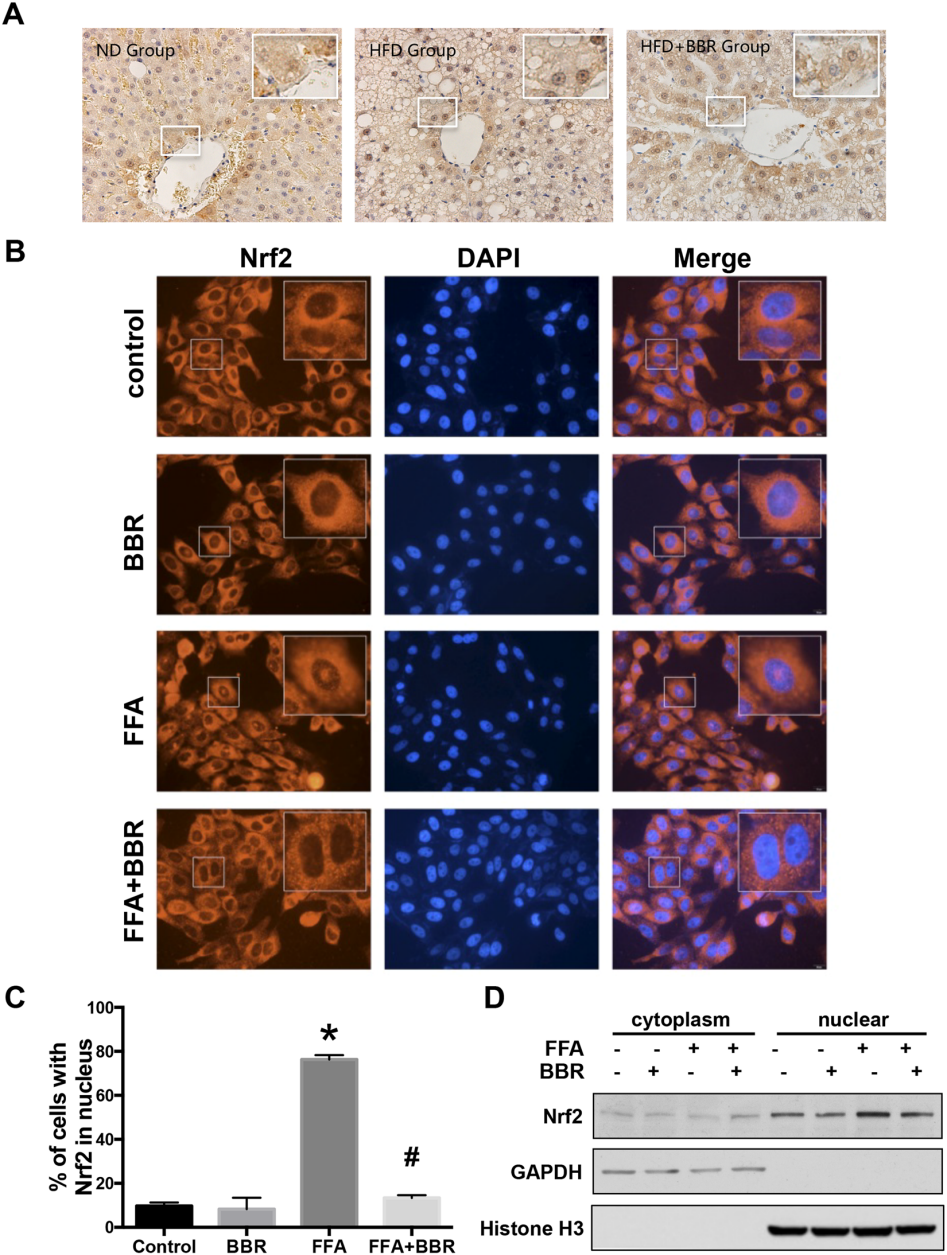
BBR inhibits Nrf2 nuclear translocation. (**A**) Representative IHC analysis of Nrf2 in the livers of HFD rats treated with BBR or vehicle for 16 weeks. (**B**) Immunofluorescence staining for Nrf2 (red) and nuclei (blue, DAPI) in Huh7 cells treated with FFA and BBR. (**C**) Quantification of cells positive for nuclear Nrf2. The average percentages of cells with Nrf2 in nucleus were quantified from a randomly selected pool of 3–6 fields in each group. (**D**) Western blots for Nrf2-enriched fractions in Huh7 cells treated with FFA and BBR. The results are expressed as the mean ± SEM. *p < 0.05 vs control, ^#^p < 0.05 vs FFA.

Nrf2 is a transcription factor that translocates to the nucleus after activation by ROS; thus, the nuclear translocation of Nrf2 reflects the cellular oxidative status. Here, we detected the distribution of Nrf2 in hepatocytes in rat liver and found that Nrf2 was primarily located in the cytoplasm of hepatocytes from the normal diet group, while it was predominantly located in the nucleus of HFD rat livers (Fig. 2A). However, the Nrf2 content in the nucleus was reduced in the livers of HFD rats that were treated with BBR versus untreated animals (Fig. 2A), suggesting that Nrf2 activation was reduced by BBR.

**Figure 3:**
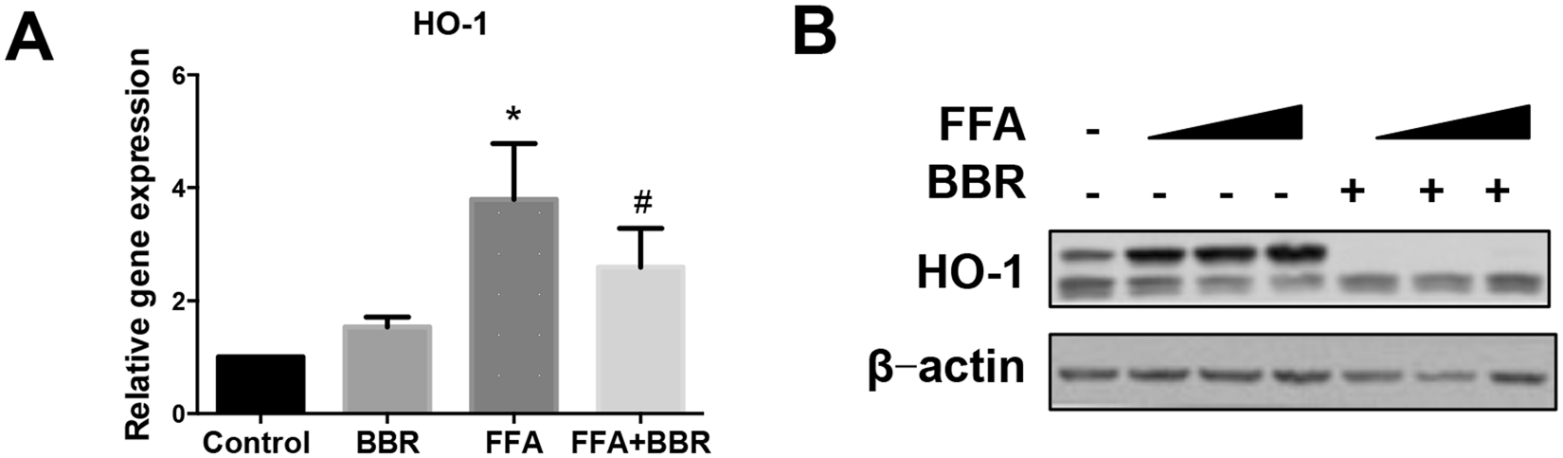
BBR down-regulates Nrf2 target gene expression. (**A**) Real-time quantitative PCR and (**B**) western blot analysis of HO-1 in Huh7 cells treated with FFA and BBR. Data are expressed as the mean ± SEM. *p < 0.05 vs control, ^#^p < 0.05 vs FFA.

In Huh7 cells, we also observed an increase in the nuclear translocation of Nrf2 with FFA treatment. However, Nrf2 nuclear translocation was markedly reduced by BBR treatment in Huh7 cells (Fig. 2B,C), which was confirmed by western blot (Fig. 2D). Immunofluorescence staining and western blot analyses were performed in Huh7 cells *in vitro*, and the results supported our findings in rat livers *in vivo*. Once Nrf2 is activated, it promotes the expression of several genes, including HO-1, which exert anti-oxidant effects by removing toxic haeme groups^6^. We also found that mRNA levels of HO-1 were significantly up-regulated by FFA treatment but suppressed by BBR (Fig. 3A). HO-1 expression was increased by FFAs in a dose-dependent manner but was completely suppressed by BBR (Fig. 3B). As shown in Fig. 3B, two bands were visualized, in which the upper band is the endogenous HO-1 protein as it was induced by FFA and repressed by administration with BBR. Notably the lower band is a non-specific band, which was visualized with long exposure time.

### BBR attenuates oxidative stress in hepatocytes

**Figure 4:**
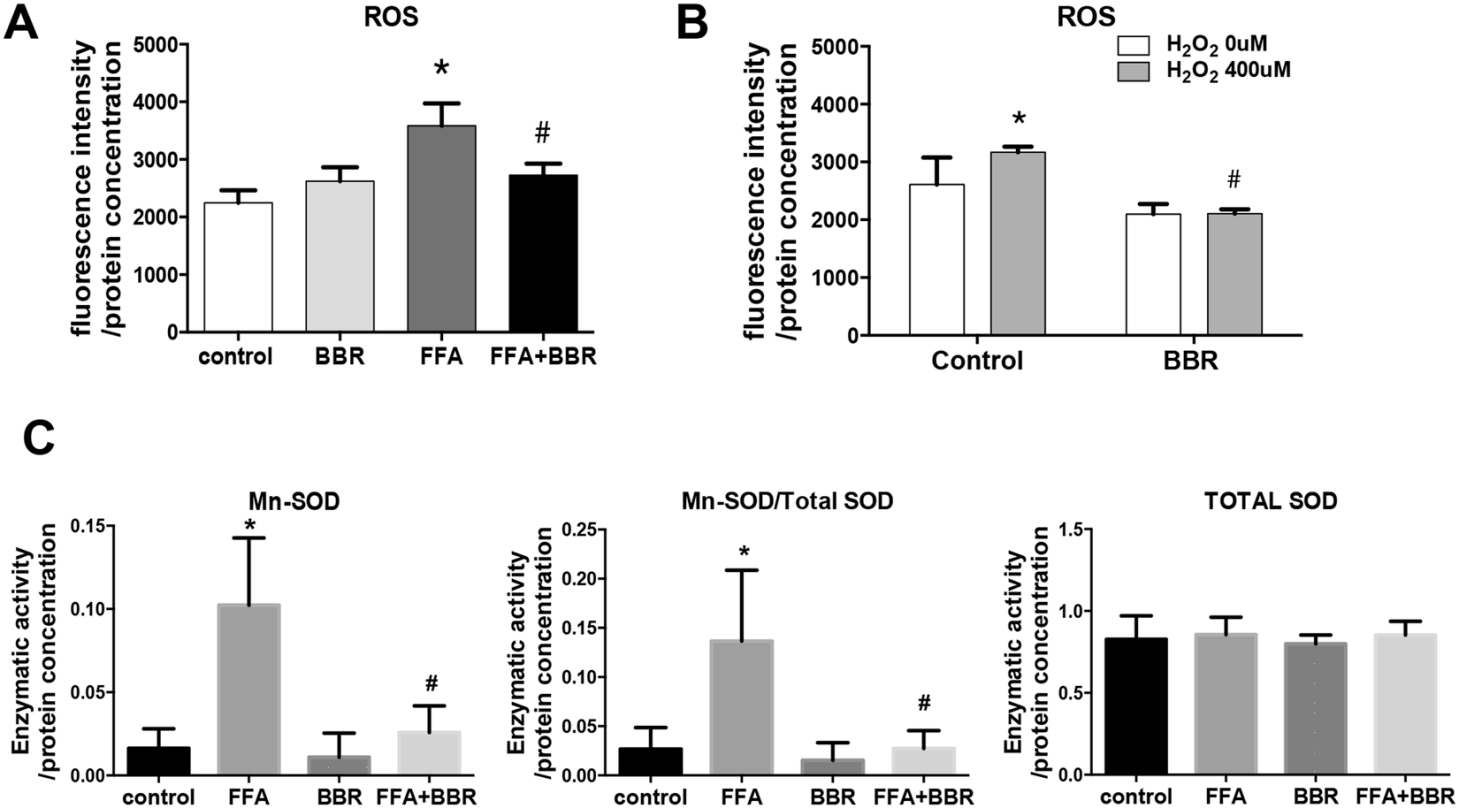
BBR reduces ROS and SOD enzymatic activities. ROS concentrations were determined using DCFH-DA in Huh7 cells treated with (**A**) FFAs at concentrations of 200 µM OA + 100 µM PA for 8 h and with or without 10 µM BBR for 24 h. Data are expressed as the mean ± SEM. *p < 0.05 vs control, ^#^p < 0.05 vs FFA, and (**B**) In the presence of H_2_O_2_ for 4 h, and treated with 10 µM BBR for 4 h. Data are expressed as the mean ± SEM. *p < 0.05 vs Control (H_2_O_2_ 0 µM); ^#^p < 0.05 vs Control (H_2_O_2_ 400 µM). (**C**) Mn-SOD activity was determined and normalized by total SOD activity. Data are expressed as the mean ± SEM. *p < 0.05 vs control, ^#^p < 0.05 vs FFA.

As oxidative stress is the stimulus that activates Nrf2 and nuclear translocation, we next investigated the effect of BBR on cellular oxidative stress. BBR treatment attenuated cellular oxidative stress in Huh7 cells stimulated by FFAs. In the presence of FFAs, cellular ROS production was dramatically increased. As shown in Fig. 4A, in cells pretreated with 10 μM BBR for 24 h, FFA-induced ROS production was completely abolished (Fig. 4A). H_2_O_2_-treated cells also displayed an increase in ROS generation and were used as positive controls (Fig. 4B). We also found that BBR treatment for 4 h significantly reduced the cellular ROS production induced by 400 µM H_2_O_2_ (Fig. 4B). As a member of the superoxide dismutase family, Mn-SOD is known to limit the detrimental effects of ROS^7^. Mn-SOD activity assays revealed that total SOD enzymatic activity was similar in cells with treated with FFA alone or FFA plus BBR. However, Mn-SOD enzymatic activity and Mn-SOD/total SOD activity were significantly increased by FFA, which was attenuated by BBR treatment (Fig. 4C).

Taken together, these findings indicate that FFA induces cellular ROS production, and Mn-SOD compensates for cellular oxidative stress. ROS generation can be reduced by BBR treatment.

### BBR reduces ROS generation in a Nox2-dependent manner

**Figure 5:**
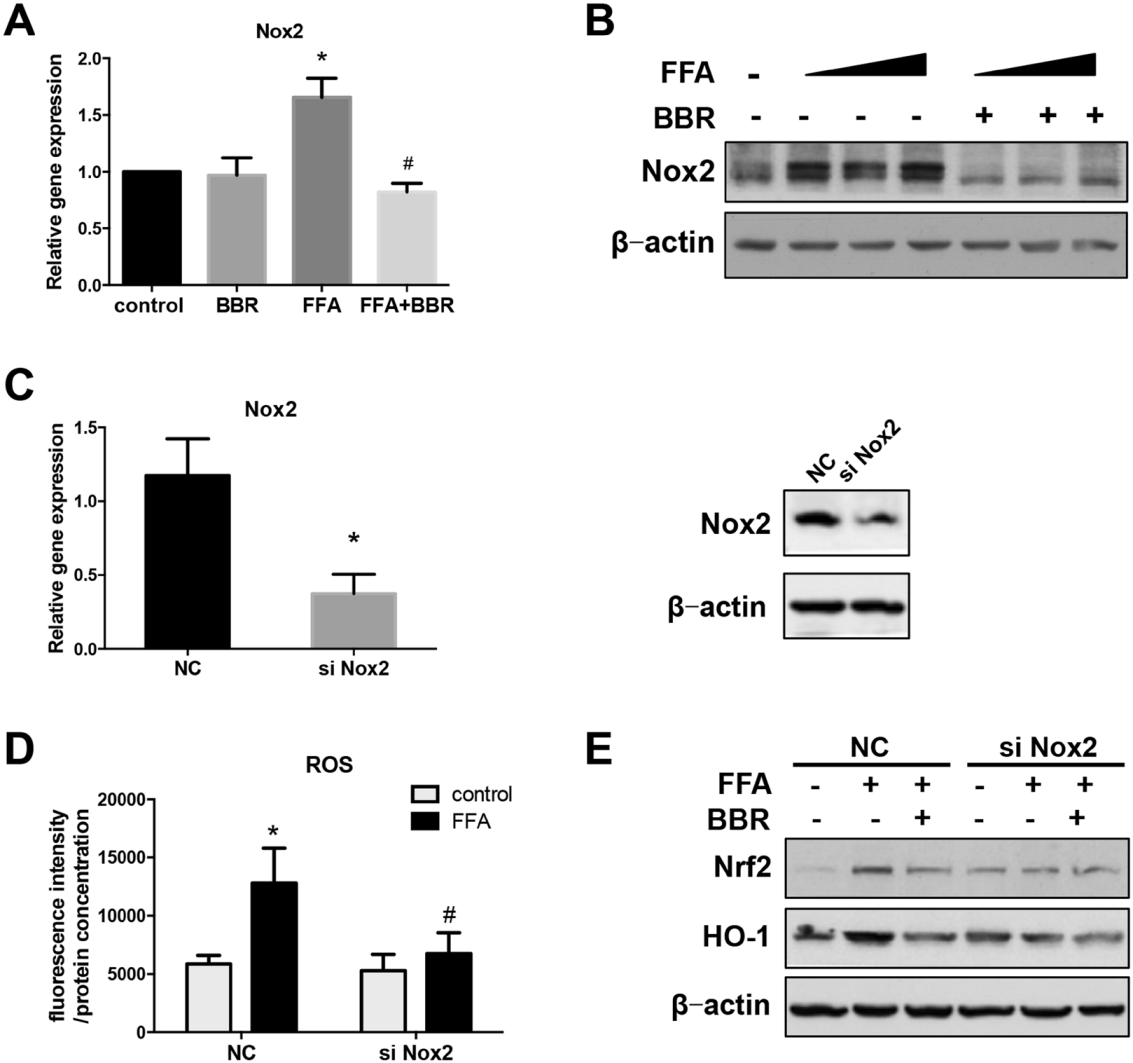
Nox2 expression and function in Huh7 cells treated with FFA and BBR. (**A**) Real time PCR analysis and (**B**) western blot of Nox2 in Huh7 cells treated with FFAs and BBR. (**C**) Real time PCR analysis and western blot of Nox2 expression in Huh7 cells transfected with siRNA targeting Nox2 or normal control (NC). (**D**) ROS production in Huh7 cells transfected with NC siRNA or Nox2 siRNA. (**E**) Immunoblots of Nrf2 and HO-1 expression in Huh7 cells treated with Nox2 siRNA. β-actin was used as a loading control. Data are expressed as the mean ± SEM. *p < 0.05 vs control, ^#^p < 0.05 vs FFA.

The NADPH oxidase (nicotinamide adenine dinucleotide phosphate oxidase, or Nox) family is dedicated to producing superoxide^8^. Nox2 is the prototypical member of this family and major isoform of NADPH oxidase^8^. Our results revealed that FFA increased the mRNA and protein expression of Nox2, which could be blocked by BBR (Fig. 5A,B). To investigate whether FFA-induced ROS generation is Nox2-dependent, we measured ROS production in Huh7 cells after FFA treatment in cells with Nox2 siRNA knockdown (Fig. 5C). Surprisingly, FFA did not significantly induce cellular ROS production in cells with Nox2 knockdown compared to normal control (NC) cells (Fig. 5D). Furthermore, and in support of a causal role of Nox2 in cellular ROS production, Nox2 knockdown also prevented FFA-induced increases in the protein levels of the anti-oxidative transcription factor Nrf2 and its downstream target HO-1 in whole cell lysates from Huh7 cells (Fig. 5E). BBR is sufficient to inhibit expression level of HO-1 although the efficacy of appears to be variable, as there was a complete suppression of HO-1 by BBR in Fig. 3B, while only a minimal reduction of HO-1 with BBR treatment under NC in Fig. 5E. The different inhibitory effects of berberine on HO-1 are likely due to many reasons, such as the condition of cultured hepatocytes as well as the protein stability of HO-1.

### BBR reduces mitochondrial-derived ROS

**Figure 6:**
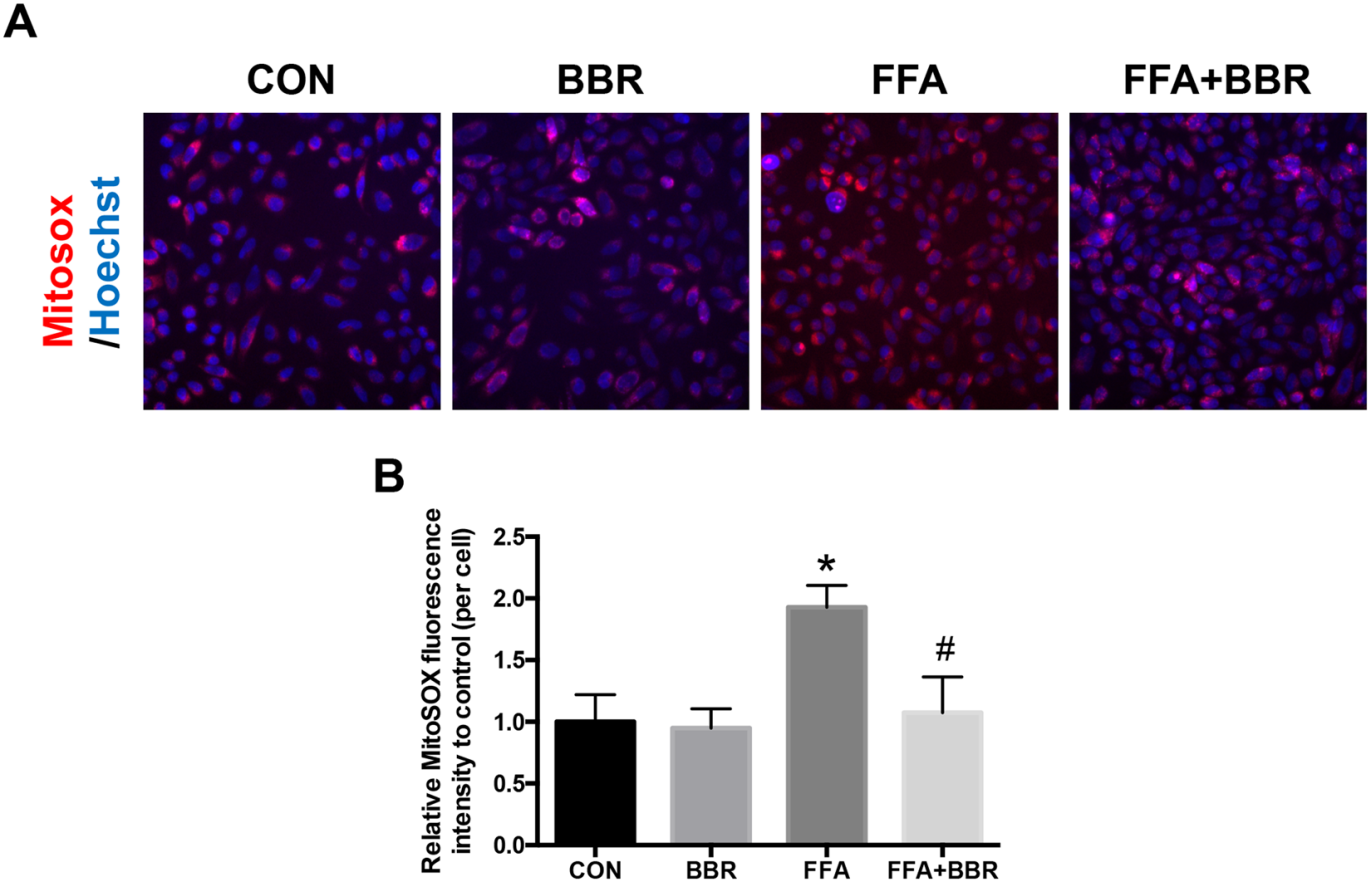
Immunofluorescence of mitochondrial ROS. (**A**) Mitochondrial ROS was determined using MitoSOX fluorescence (red). Hoechst 33342 (blue) was used to detect the nucleus. (**B**) MitoSOX fluorescence intensity was quantified as integrated intensity per cell versus control. *p < 0.05. Data are expressed as the mean ± SEM. *p < 0.05 vs control, ^#^p < 0.05 vs FFA.

Our data indicated that BBR reduced FFA-induced ROS in a Nox2-dependent manner. However, the major source of intracellular ROS is the mitochondria^9^. Therefore, we measured mitochondrial ROS in Huh7 cells using MitoSOX Red, a mitochondrial superoxide indicator^10^. We found that the MitoSOX Red fluorescence (red) levels in the cytoplasm were increased after FFA treatment (Fig. 6). BBR markedly reduced the cellular MitoSOX Red fluorescence in cells treated with FFA (Fig. 6). These data suggest that BBR has the capacity to reduce mitochondrial-derived ROS production induced by FFA treatment.

### The effects of BBR on mitochondrial ETC complex and respiration

**Figure 7:**
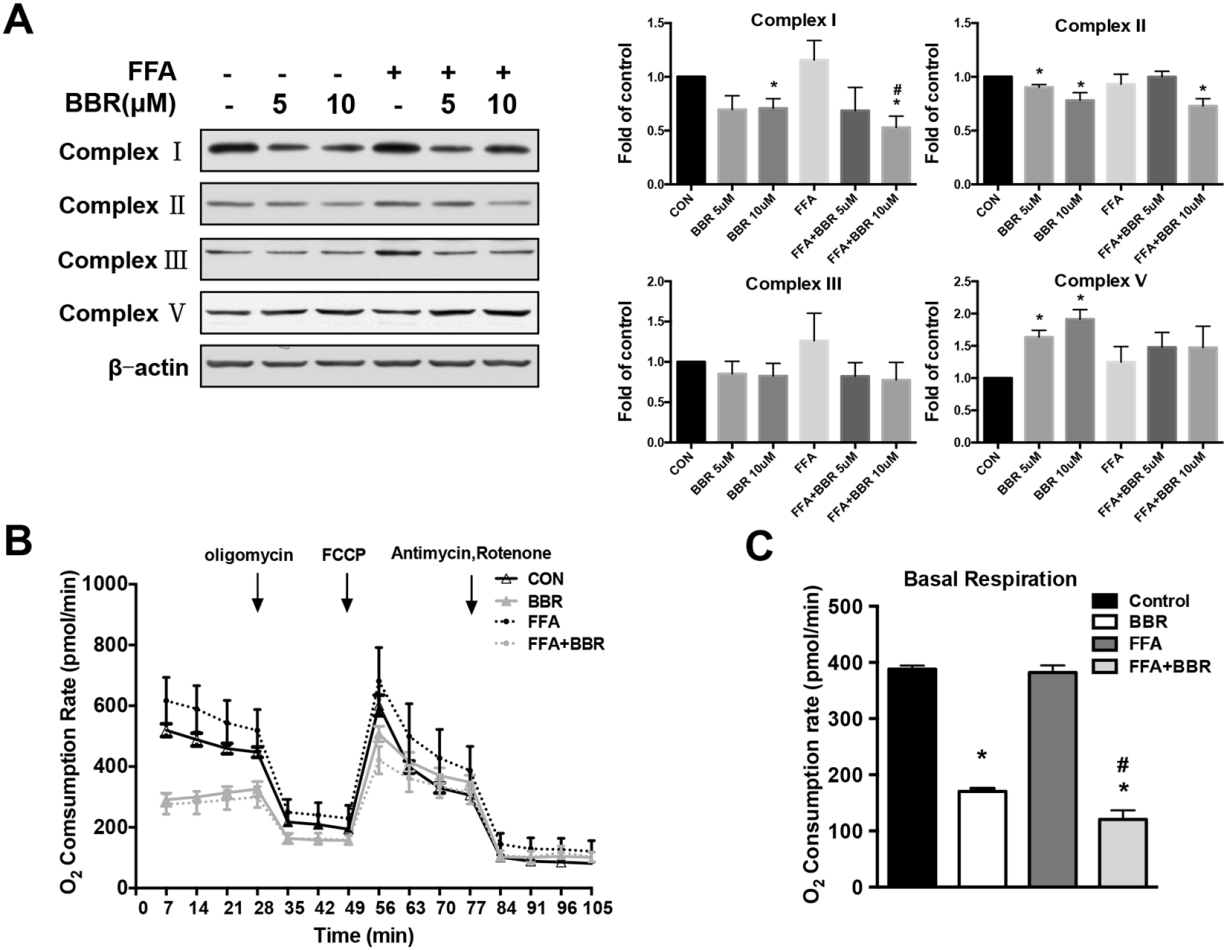
The change in mitochondrial electron transport chain complex expression and oxygen consumption by BBR. (**A**) A representative western blot for electron transport chain complex I, II, III, and V in Huh 7 cells treated with FFAs and BBR. The densitometric units of each protein band were calculated from three independent experiments versus the control group. (**B**) The oxygen consumption rate was determined by Seahorse XF-24 in HepG2 cells treated with FFAs and BBR in response to mitochondrial compounds, including oligomycin, FCCP, antimycin, and rotenone. (**C**) Oxygen consumption rates of basal respiratory capacity were calculated from the real-time oxygen consumption curve presented in (**B**). *p < 0.05. Data are expressed as the mean ± SEM. *p < 0.05 vs control, ^#^p < 0.05 vs FFA.

The mitochondrial electron transport chain (ETC) plays a major role in the generation of ROS. A fraction of electrons being transported by the ETC leak from complex I and III to form superoxide anion radicals. The excess deposition of lipids within hepatocytes provides an excess fuel supply for the mitochondrial oxidation reaction, which leads to copious ROS production^11^, resulting in oxidative stress. We then studied the effects of BBR on the expression of the ETC complex in Huh7 cells. We found that the expression of complexes I and III was slightly increased by FFA compared with controls. BBR treatment reduced the expression levels of complex I, II, and III, especially complex I and II (Fig. 7A). Thus, ROS produced by the mitochondrial ETC was reduced. However, complex V (ATP synthase) expression was markedly increased by BBR treatment (Fig. 7A), indicating that BBR did not inhibit the ATP synthetic functions of the mitochondria. Using the Seahorse XF-24 analyser, we next determined the mitochondrial oxygen consumption rate (OCR) of HepG2 cells in real time (Fig. 7B). Our results showed a decreased basal respiration capacity in response to BBR treatment (Fig. 7C).

## Discussion

In the present study, we confirmed that BBR has the capacity to reduce lipid accumulation in the livers of mice fed a HFD. Interestingly, the results in this study also demonstrated that the nuclear distribution of the anti-oxidant transcription factor Nrf2, as well as the transcription of its downstream target gene, HO-1, was reduced by BBR treatment. To investigate the reason for the difference in Nrf2 activation, we measured ROS generation and found that cytoplasmic ROS production induced by FFA was attenuated by BBR in a Nox2-dependent manner. Meanwhile, mitochondrial-derived ROS production was abrogated by BBR treatment. This ultimately resulted in a lower state of oxidative stress in the cells, leading to reduced Nrf2 nuclear translocation in response to BBR treatment.

### Regulation of Nrf2 activation by berberine

Either reducing ROS generation or promoting ROS clearance can protect cells against the damage from oxidative stress. Nrf2 is an emerging regulator of anti-oxidant stress, as it regulates a number of genes responsible for anti-oxidative stress^12^. Activation of the Nrf2 pathway can promote ROS clearance. Most proteins encoded by downstream target genes of Nrf2 could ameliorate oxidative stress. For example, Nrf2 induces the catabolism of superoxide and peroxides through its target gene products—SOD, peroxiredoxin and glutathione peroxidase^12^. Nrf2 is usually located in the cytoplasm bound to Kelch-like erythroid cell-derived protein with CNC homology-associated protein 1 (Keap1)^13^. When challenged with ROS, Nrf2 dissociates from Keap1 and trans-locates into the nucleus, which triggers the expression of its target genes^14^. As an anti-oxidant transcription factor, Nrf2 distribution in nucleus indicates the activity of Nrf2.

The results of our study showed that BBR ameliorates oxidative stress by reducing ROS formation. In the presence of FFA, cellular ROS production was dramatically increased, but in cells pretreated with BBR, FFA-induced ROS production was completely abolished. Nrf2 is activated by oxidative stress, demonstrating its anti-oxidant potential, and protects cells from oxidative damage^15^. In response to a low oxidative stress status, Nrf2 continues binding to Keap1 in the cytoplasm and does not trans-locate into the nucleus. *In vivo* and *in vitro* approaches were applied in our study to indicate that Nrf2 entry into the nucleus is reduced after BBR administration compared to that in HFD-induced liver or FFA-stimulated hepatocytes. First, IHC results showed that Nrf2 was located mainly in the cytoplasm of BBR-treated rat livers, while it was distributed predominantly in the nucleus in the HFD only group. Second, an increase of Nrf2 distribution in the nucleus was also observed in FFA-stimulated Huh7 cells but not in cells treated with BBR. Furthermore, after separating the cytoplasmic and nuclear fractions, the western blotting results showed that Nrf2 expression in the nucleus was higher with FFA induction, which did not occur in cells co-treated with FFA and BBR. Our results indicated that the inhibition of ROS formation by BBR contributed to decreased activation of Nrf2, resulting in its reduced nuclear distribution. BBR showed its anti-oxidative stress potential by inhibiting ROS generation; thus, the demand for anti-oxidative stress activity was reduced. These results are consistent with the observations in the study of Sengupta D *et al*.^16^. They found that carcinogen-increased ROS generation in hepatocytes was accompanied by a 3.3-fold higher Nrf2 expression in the nucleus compared with the control. BBR reduced ROS content induced by diethylnitrosamine and CCl_4_ exposure, but nuclear Nrf2 expression remained 2.03-fold higher than that in the control^16^. Sengupta D *et al*. indicated that berberine maintained activation of the Nrf2/HO-1 pathway during the treatment period, showing the anti-oxidative effect of berberine. However, we found that the nuclear expression of Nrf2 in the berberine-treated group was 2.03-fold higher than that in the control, but it was 1.27 (=3.3-2.03)-fold less than that in the carcinogen-exposed group in their study. Their results support our findings, in our data, FFA promoted ROS accumulation, and the increased Nrf2 in the nucleus in response to FFA indicated up-regulated activity of the anti-oxidant transcription factor Nrf2. As ROS accumulation mediated maintenance of the Nrf2/HO-1 pathway^16^, berberine reduced FFA-induced ROS accumulation, which resulted in a lower activation status of Nrf2, as well as a decreased Nrf2 in the nucleus compared with that in FFA-stimulated cells.

However, in Yuan’s study, Nrf2 mRNA was decreased in HFD-fed rat liver, but this change was significantly reversed by berberine treatment^17^. In our *in vitro* study, we found that FFA increased the protein level of Nrf2, which was reversed by berberine treatment. Sengupta D *et al*. also revealed a decrease in Nrf2 protein expression level in the berberine treatment group compared with that in the carcinogen-exposed group^16^, which was consistent with our findings. The discrepancy might be due to the different model we used. In Yuan’s study, they conducted the real-time PCR with liver samples from long-term HFD-fed rats, with or without BBR treatment. In our study, we performed western blotting with cell lysate samples from Huh7 cells treated with FFA and/or berberine for a short time. The different models and different induction time may contribute to the inconsistency between their results and ours. However, the mechanism underlying this discrepancy is still not clear and needs to be explored.

Due to reduced Nrf2 nuclear translocation, the transcription of the Nrf2 downstream gene HO-1 was also decreased after BBR treatment, which was confirmed by our results. According to a previous study, increased HO-1 protein levels, due to Nrf2 activation, promoted insulin resistance and inflammation^18^, which suggests that increased activation of anti-oxidative stress effects may also damage the cell. Cellular homeostasis of the anti-oxidative stress system is necessary. BBR reduces the activation of the Nrf2/HO-1 pathway by inhibiting ROS formation, which contributes to a balanced homeostasis of the anti-oxidative stress system. Therefore, to some degree, the inhibition of Nrf2 activation and HO-1 expression by BBR may mediate its role in improving metabolic disease.

### Inhibition of ROS generation by berberine

The association of ROS production and fatty liver disease has been well studied^19, 20^. In obese animals or humans, lipolysis in adipose tissues is substantially increased due to insulin resistance. Excessive lipid uptake by the liver provides surplus substrates for oxidation, contributing to heightened ROS formation^21^. Accumulating amounts of ROS further damage the liver, causing lipid peroxidation and cytokine release^22^, which promotes hepatocyte inflammation and apoptosis.

Cytoplasmic ROS production is influenced by the NADPH oxidase family, which couples electrons transferred across the cell membrane to oxygen, thus generating ROS^8^. NADPH oxidase 2 (Nox2) is a potent source of cytoplasmic ROS generation^23^. Nox2 expression was correlated with the severity of hepatic steatosis in patients with NAFLD^24, 25^. We revealed that ROS generated in response to FFA is mediated by Nox2, while BBR could reverse FFA-induced Nox2 expression. Our results demonstrated that Nox2 may be essential for BBR to reduce ROS formation.

The mitochondrion is the powerhouse of the cell, which supplies ATP and energy. However, the mitochondrion is also a major source of ROS, while energy substrates are interchangeable, including glucose and triglyceride^1^. Using MitoSOX Red staining, which is a mitochondrial superoxide indicator, we found that BBR reduces the mitochondrial-derived ROS production induced by FFA. In the process of electron transport within the mitochondrial inner membrane, ROS are generated as a by-product of respiration via electron leakage in complex I and III^26^. A previous study by Fernyhough *et al*. demonstrated that the decreased protein expression of members of the respiratory chain occurs in the axons of diabetic rats and is correlated with decreased intra-mitochondrial superoxide generation derived from the mitochondrial respiratory chain^27^. Our results showed that BBR attenuated mitochondrial ROS and down-regulated complex I and III expression. At the same time, the expression of complex V was increased after BBR treatment, indicating that mitochondrial ATP production is not reduced by BBR treatment, since complex V serves as an ATP synthase^28^. Furthermore, using Seahorse XF-24 assays, we found that the basal oxygen consumption rate in HepG2 cells treated by BBR was significantly reduced. These results suggest that BBR inhibits ROS generation from the mitochondrion by down-regulating the expression of major leakage site—complex I—and reducing the material—oxygen—for ROS formation.

Anti-oxidants have been applied for therapy of liver diseases. For example, vitamin E attenuates oxidative damage by scavenging reactive oxygen species^29^. Carotenoids display anti-oxidant effects by quenching singlet oxygen^30^. In our study, BBR served as a promising anti-oxidant by inhibiting the generation of ROS.

In conclusion, BBR improves the state of oxidative stress in hepatocytes, which possibly contributes to reducing the risk of NASH progression or even cirrhosis.

## Materials and Methods

### Animals

Seven- to eight-week-old male C57BL/6 mice were purchased from the Shanghai Laboratory Animal Company in China. Five- to six-week-old male Sprague Dawley (SD) rats were purchased from the Animal Development Centre, Chinese Academy of Science, Shanghai.

C57BL/6 mice were fed a normal chow diet or high fat diet (HFD; D12492, Research Diet, USA) for 8 weeks, after which time they were given BBR (300 mg/kg/d, Sigma, St. Louis, USA) or vehicle orally for another 8 weeks. Rats were fed a normal diet (ND, 62.3% carbohydrate, 12.5% fat and 24.3% protein in total calories) or HFD (32.6% carbohydrate, 51.0% fat and 16.4% protein) for 8 weeks and were then gavaged with BBR (200 mg/kg/d) or vehicle for the next 16 weeks. Animals were housed at constant room temperature under a 12 h light/dark cycle with *ad libitum* access to water and chow. All animal procedures were approved by the Institutional Animal Care and Use Committee (IACUC) of Fudan University. All experiments were performed in accordance with the relevant guidelines and regulations.

### Measurement of liver triglycerides

Mouse liver tissues were homogenized with PBS, followed by extraction with a mixture of chloroform and methanol at a ratio of 2:1. After drying under nitrogen gas, the lipid was then resuspended in ddH_2_O. Liver triglyceride (TG) levels were determined using a triglyceride kit (Shensuoyoufu, China) and normalized to total protein concentration.

### Histological and immunohistochemistry analysis

Rat liver tissue was fixed in phosphate-buffered 10% formalin, processed for histological assessment, and stained with haematoxylin-eosin using standard procedures. Immunohistochemistry was performed on paraffin-embedded sections of rat liver tissue. After deparaffinization, rehydratation, and antigen retrieval, sections were further incubated with primary anti-Nrf2 antibody (Abcam, Cambridge). After incubation with secondary antibody and staining with diaminobenzidine (DAB), the sections were counterstained with haematoxylin, dehydrated, and sealed with a coverslip. Images were captured with an Olympus microscope (Olympus Corporation, Japan).

### Cell culture

The human hepatoma cell lines Huh7 and HepG2 were maintained in Dulbecco’s modified Eagle’s medium (DMEM) supplemented with 10% (v/v) foetal bovine serum and 1% (v/v) penicillin/streptomycin in an atmosphere of 5% CO_2_ and 37 °C. For free fatty acid (FFA) treatment, a stock solution of 10 mM oleic acid (OA) or 10 mM palmitic acid (PA) was prepared in 5% (w/v) fatty acid free-bovine serum albumin (BSA). Huh7 and HepG2 cells were grown to 70% confluence and exposed to a FFA mixture containing OA and PA at a molar ratio of 2:1 in the absence or presence of BBR. BBR (Sigma, USA) was dissolved in dimethylsulfoxide (DMSO) (Sigma, USA) to a stock concentration of 20 mM and was filtered and stored at −20 °C prior to use.

### Nile Red staining

Huh7 cells affixed to cover slips were rinsed quickly with 0.01 M PBS and fixed with 4% paraformaldehyde for 15 min. After washing twice with 0.01 M PBS, cells were incubated with 0.75 µg/ml Nile Red for 15 min at room temperature. Cells were then counterstained with 100 ng/ml DAPI at room temperature for 5 min. After washing, the cover slips were then mounted.

### Immunofluorescence staining

Cells were rinsed quickly with 0.01 M PBS and blocked in 10% goat serum at room temperature for 1 hour. Then, cells were incubated overnight at 4 °C with primary antibody (rabbit anti-Nrf2). After rinsing with 0.01 M PBS three times, the cells were incubated with Alexa Fluor 488 donkey anti-rabbit IgG overnight at 4 °C. Cells were then counterstained with DAPI for 1 min. After washing, the stained cells were examined by an Olympus microscope (Olympus Corporation, Japan). The average percentages of cells with Nrf2-positive nuclei in the immunofluorescence staining results were quantified from a randomly selected pool of 3–6 fields under each condition. The data are presented as “% of cells with Nrf2 in nucleus”.

### ROS detection

ROS generation by Huh7 cells was detected with the fluorescent probe 2,7-dichlorofluorescein diacetate (DCFH-DA; Beyotime Institute of Biotechnology, China). After stimulation with FFA or BBR, cells were rinsed with PBS and incubated with 10 µM DCFH-DA for 20 min at 37 °C. This fluorescent probe diffuses into cells and is metabolized into a highly fluorescent compound, 2,7-dichlorofluorescein (DCF), by intracellular H_2_O_2_ or OH in the presence of peroxidase. ROS production was measured by fluorescence intensity using a fluorescence microplate reader (PerkinElmer, CA, USA), which was normalized to total protein content (in each well).

### Mn-SOD activity measurement

FFA- and BBR-treated Huh7 cells were rinsed and lysed in cold PBS. After centrifugation at 4 °C, supernatants were collected and diluted with SOD measurement buffer and were then used for a SOD enzyme activity assay following the manufacturer’s instructions (Beyotime Institute of Biotechnology, China). Briefly, in a 96-well plate, 160 μl WST-8/enzyme working solution and 20 μl reaction working solution were mixed with 20 μl cell lysates (acquired from the above procedures) or SOD measurement buffer; the latter was defined as Blank 1. One hundred sixty μl WST-8/enzyme working solution combined with 40 μl SOD measurement buffer was used as Blank 2. The above mixtures were incubated at 37 °C for 30 min, and absorbance values at 450 nm were determined. Total SOD enzymatic activity was calculated by the following equations and normalized to the protein content of each sample: (1) inhibition ratio = (absorbance Blank1-absorbance Sample)/(absorbance Blank1-absorbance Blank2) * 100% and (2) SOD enzymatic activity units = inhibition ratio/(1-inhibition ratio). To determine Mn-SOD activity, the Cu/Zn-SOD inhibitor A and sample were mixed at a ratio of 1:24 and then incubated at 37 °C for 1 h, yielding Mix 1. Cu/Zn-SOD inhibitor B was diluted by a factor of 40 and combined with Mix 1 at a ratio of 1:25 and then incubated at 37 °C for another 15 min. To determine the Mn-SOD enzymatic activity, we followed the same procedure as the one for Total SOD enzymatic activity detection.

### siRNA transfection

Huh7 cells were seeded into 6-well plates, and the media were replaced with Opti-MEM (Gibco, USA) once the cells had grown to 70% confluence. Two hundred µl Opti-MEM was mixed with either 2.5 µl siRNA (20 µM) or 5 µl Lipofectamine 2000 (Invitrogen, USA) to make Mixture 1 and Mixture 2, which were incubated at room temperature for 5 min. The above two mixtures were then mixed together gently and incubated at room temperature for another 20–25 min. Cell media was replaced by the above mixture in 2 ml/well cell media. The transfection duration was 48 h. siRNA sequences were as follows, Nox2 sense 5′-CUGUGAUAAGCAGGAGUUUdTdT, Nox2 antisense 5′-AAACUCCUGCUUAUCACAGdTdT, normal control (NC) sense 5′-UUCUCCGAACGUGUCACGUdTdT, and normal control (NC) antisense 5′-ACGUGACACGUUCGGAGAAdTdT.

### Real-time polymerase chain reaction

Total RNA from cultured cells was isolated and converted to complementary DNA. The messenger RNA (mRNA) expression levels of target genes were determined by real-time polymerase chain reaction (real-time PCR) using an Applied Biosystems 7500 (Applied Biosystems, USA) with SYBR Premix EX Taq (TaKaRa, Japan). The sequences of primers used are shown in Supplemental Table 1. β-actin gene expression was used as an internal control.

### Western blotting

Huh7 cells were lysed in RIPA buffer containing protease and phosphatase inhibitors. After being boiled, proteins were loaded in SDS-PAGE and blotted onto a polyvinylidenedifluoride membrane and were detected using specific antibodies against Nrf2 (Abcam, Cambridge), HO-1 (Proteintech), Nox2 (Proteintech), total OXPHOS (oxidative phosphorylation) antibody cocktail (Abcam, Cambridge), GAPDH (Sigma-Aldrich, USA), Histone H3 (Cell Signaling Technology, USA) and β-actin (Santa Cruz, USA). Membranes were then washed in TBST and incubated with goat anti-rabbit secondary antibody (Santa Cruz, USA) for 1 h before being visualized.

### Intramitochondrial ROS measurement

For detection of intramitochondrial ROS, mainly superoxide, 5 µM MitoSOX Red reagent (Molecular Probes Invitrogen, USA) working solution was applied to cover cells adhering to cover slips and then incubated for 10 minutes at 37 °C while protected from light. After washing with warm buffer, cells were then counterstained with 1 µg/ml Hoechst 33342 (Thermo Scientific, USA) for an additional 10 min at 37 °C. The stained cells were examined by an Olympus microscope (Olympus Corporation, Japan).

### Respiration measurement

Hepatocytes were seeded into XF24 cell culture plates. After drug treatment (FFA or BBR), the media were removed, and cells were washed twice with warm Seahorse assay medium (Agilent Technologies, USA) and incubated for 1 h at 37 °C without CO_2_. The compounds oligomycin, FCCP, antimycin, and rotenone of the Seahorse XF Cell Mito Stress Test Kit (Agilent Technologies, USA) were diluted in Seahorse assay medium and were preloaded into reagent delivery ports of A, B, and C of the O_2_ sensor cartridge. Oxygen consumption rate (OCR) measurements were then carried out according to the Seahorse assay protocol using a Seahorse XF-24 analyser (Agilent technologies, USA). The basal level of OCR was measured 4 times, and the maximal respiratory capacity was determined by OCR after FCCP injection, minus the one before oligomycin injection.

### Statistical analysis

All data are expressed as the mean ± SEM. Statistical significance was assessed using a two-tailed Student *t* test. Differences between treatments at P < 0.05 were defined as statistically significant.

## Electronic supplementary material

The primers of genes for real time-qPCR
