# Supplementary material for: Berberine ameliorates fatty acid-induced oxidative stress in human hepatoma cells
Source: Sci Rep. 2017 Sep 12;7:11340. doi: 10.1038/s41598-017-11860-3 (PMC5595831; doi:10.1038/s41598-017-11860-3)
Supplement: Supplementary file 1 — The primers of genes for real time-qPCR [file 41598_2017_11860_MOESM1_ESM.doc]

**Berberine ameliorates fatty acid induced oxidative stress in human hepatoma cells**

Yixuan Sun^1，2#^, Xinlu Yuan^1#^, Feifei Zhang^3^, Yamei Han^3^, Xinxia Chang^1^, Xi Xu^1^, Yu Li^3^, Xin Gao^1，2*^

^1^Department of Endocrinology and Metabolism, Zhongshan Hospital, Fudan University, Shanghai 200032, China; ^2^ Fudan Institute for Metabolic Diseases, Shanghai 200032, China ；^3^ CAS Key Laboratory of Nutrition and Metabolism, Institute for Nutritional Sciences, Shanghai Institutes for Biological Sciences, University of Chinese Academy of Sciences, Shanghai 200031, China

^#^These authors contributed equally to this work .

*Corresponding to:

Xin Gao, MD

Professor

Department of Endocrinology and Metabolism, Zhongshan Hospital,

Fudan Institute for Metabolic Diseases,

Fudan University,

180 Feng Lin Road，

Shanghai 200032, China

Email: [happy20061208@126.com](mailto:happy20061208@126.com)

**Supplemental Table 1. The primers of genes for real time-qPCR**

| Gene | Strand | Primer (5’-3’) |
| --- | --- | --- |
| Nfe2l2(Nrf2) | sense | GCCGAAGAAACCTCATTGTC |
|  | antisense | GCCGAAGAAACCTCATTGTC |
| HO-1 | sense | CCTGCTCAACATCCAGCTCT |
|  | antisense | TCTTGCACTTTGTTGCTGGC |
| 18S | sense | GCTGGAATTACCGCGGCT |
|  | antisense | CGGCTACCACATCCAAGGAA |
